# Treatment of Anxiety Disorders and Comorbid Alcohol Abuse with Buspirone in a Patient with Antidepressant-Induced Platelet Dysfunction: A Case Report

**DOI:** 10.1155/2013/572630

**Published:** 2013-12-12

**Authors:** Mir Mazhar, Tariq Hassan, Tariq Munshi

**Affiliations:** Department of Psychiatry, Queen's University, Kingston, ON, Canada K7L 7X3

## Abstract

The risk of abnormal bleeding with serotonin reuptake inhibitors has been known, but there is insufficient evidence base to guide pharmacological treatment of anxiety in patients with underlying haematological conditions. The following case report is about a 50-year-old female with generalized anxiety disorder, social phobia, obsessive compulsive disorder, and alcohol abuse where pharmacological treatment of anxiety symptoms has been difficult as it would lead to bruising due to the patient's underlying qualitative platelet dysfunction. Treatment with venlafaxine, citalopram, escitalopram, and clomipramine resulted in improvement and anxiety symptoms, as well as reduction in alcohol use, but pharmacological treatment has to be discontinued because of bruising and hematomas. In view of an active substance use disorder, benzodiazepines were avoided as a treatment option. The patient's anxiety symptoms and comorbid alcohol abuse responded well to pharmacological treatment with buspirone which gradually titrated up to a dose of 30 mg BID. Patient was followed for around a six-month period while she was on buspirone before being discharged to family doctor's care. Buspirone is unlikely to have a significant effect on platelet serotonin transponder and could be an effective alternative for pharmacological treatment of anxiety in patients with a bleeding diathesis.

## 1. Introduction

The risk of abnormal bleeding with serotonin reuptake inhibitors (SSRIs) has been known since a landmark British study demonstrated the association between upper gastrointestinal bleeding and SSRIs [1]. There has been evidence to suggest that antidepressant use can lead to hospitalization for abnormal bleeding. The risk of bleeding is directly correlated with the affinity of the antidepressant for the serotonin transporter, with adjusted odd ratios varying from 9.4 for Clomipramine to 1.8 for Maprotiline [2]. The risk of bleeding is increased if there is concomitant use of SSRIs with antiplatelet drugs [3]. Blockade of serotonin reuptake by platelets, leading to subsequent depletion interfering with platelet aggregation, has been postulated to increase the risk of abnormal bleeding [4]. The duration of SSRI treatment leading to bleeding such as hematemesis, epistaxis, and petechiae has been found to be between 26 and 40 days [5]. There is insufficient evidence base to guide the pharmacological treatment of anxiety in a patient with underlying haematological conditions resulting in platelet dysfunction, which makes the following case significant.

## 2. The Case

A 50-year-old divorced Caucasian female, who is the mother of two grown-up children and had been working as a government employee for 27 years, presented with anxiety symptoms first noticed when the patient was around 9 years old. The patient complained of excessive worrying about “something dreadful happening all the time,” feeling tense, inability to relax, and irritability. These psychological symptoms of anxiety were accompanied by neck and shoulder muscle aches, teeth clenching, sleep difficulties mainly consisting of initial insomnia, and impaired concentration.

The patient also reported longstanding anxiety in social settings where the patient described feeling “self-conscious” and scrutinized. The patient reported having a fear of public speaking and described significant anxiety including palpitations, sweating, and “butterflies in stomach” when she had to speak in a staff meeting. In addition, the patient avoided eating out in restaurants unaccompanied due to anxiety.

The patient also reported having a compulsion to do things in a certain order (e.g., she had to open window curtains first, let the dogs out, turn on the radio, and then have her breakfast). The patient had an underlying fear that if the order was not followed, then “something will go wrong, I will be testing fate.” The patient would avoid stepping on the cracks while walking on the pavement as she was concerned this would result in harm befalling on her family. The patient also felt compelled to count the holes in the ceiling as she believed this would protect her family from harm. These symptoms were egodystonic; the patient felt distressed by them and unable to resist them.

Since the age of 14, the patient reported consuming alcohol on a regular basis. She was presently drinking 2-3 glasses of wine every night, but there were occasions where the patient would drink excessively leading to recurrent blackouts. The patient acknowledged that the drinking leads to relationship difficulties but denied any impairment at work. The patient acknowledged her drinking to be a problem and was receiving addiction counselling.

Around the age of 44, the patient reported a suicide attempt by means of drug overdose following the breakup of her marriage. The patient was treated for depression by her family doctor with venlafaxine. The patient denied any in-patient psychiatric treatment.

The patient had positive family history, among first-degree relatives, for depression, bipolar disorder, and attention deficit hyperactivity disorder.

The patient was treated with thyroxine for hypothyroidism. The patient's thyroid stimulating hormone was within the normal range. Of note in her past medical history was bruising resulting from treatment with venlafaxine. The patient was seen by a hematologist around that time. She had tested negative for Von Willebrand's disease, Factor VIII, IX and X deficiencies. The patient tested negative for anti-cardiolipin and antinuclear antibodies. The patient's INR and platelet count were within the normal range. The patient's platelet aggregation studies suggested a qualitative platelet defect due to “absence of release of ADP and Epinephrine.” It was suggested in the hematologist's report that the venlafaxine was contributing to the platelet dysfunction, resulting in bruising. It was unclear per the report if this effect on platelet aggregation was confined to venlafaxine or if other antidepressants can cause the same effect.

A diagnosis of generalized anxiety disorder, social phobia, obsessive compulsive disorder, and alcohol abuse was made per the DSM-IV TR criteria. In terms of pharmacological interventions, the patient was started on citalopram 20 mg daily. When the patient was reviewed after four weeks, she described significant improvement in her anxiety symptoms and was feeling “very calm.” The patient has also managed to reduce her alcohol consumption from 2 to 3 glasses of wine per day to the same amount on a weekly basis. The patient reported headaches but had not noticed any bruising. Two weeks later, the citalopram was discontinued by the patient's family physician due significant bruising, and the patient was switched to escitalopram 10 mg daily. The patient remained on escitalopram for around four weeks before its discontinuation, as the bruising was spreading all over patient's body. The patient did notice continued improvement in anxiety and was able to quit drinking alcohol. The patient was given a medication-free period of two weeks during which time the patient's bruising subsided. She was then started on clomipramine 25 mg BID to see if she would respond any differently to tricyclics compared to the newer antidepressant medications. Even on a relatively low dose of clomipramine, the patient reported a reduction of her anxiety symptoms without a recurrence of bruising when reviewed in the clinic 3 weeks later. When the patient was reviewed in the clinic four weeks later, she was again all covered with bruises and had also developed a subgluteal hematoma following a fall. The patient's anxiety symptoms and alcohol abuse were still in remission. The clomipramine was immediately discontinued and the patient was advised to remain medication free for two weeks in order for the bruising to subside. The patient was concerned that she would be likely to relapse on alcohol as a self-medication strategy if her anxiety symptoms were to recur. She was started on buspirone 10 mg BID and was followed up initially on a fortnightly basis. Buspirone was considered a safer anxiolytic option as compared to benzodiazepines in view of patient's active addiction to alcohol. The patient's anxiety symptoms showed a response to buspirone, but the dose had to be gradually increased to 30 mg BID to treat residual anxiety. The patient tolerated the medication well without any recurrence of bruising. The patient was followed up in the clinic for around six months while on buspirone before being discharged to her family doctor. During this time, the patient's anxiety symptoms and alcohol abuse remained in remission.

## 3. Discussion

The average duration of antidepressant use that leads to bleeding is consistent with current evidence. Buspirone's suggested mechanism of action involves partial agonist activity at 5-HT1a receptors linked negatively to adenyl cyclase [6] and is unlikely to have any significant action on the platelet serotonin transponder. Buspirone is indicated for treatment of generalized anxiety disorder. Buspirone also has some evidence as augmentation strategy with selective serotonin reuptake inhibitors for treatment of major depressive disorder [7]. Buspirone could be an effective alternative for the treatment of anxiety in cases where there is concern about the risk of bleeding. While the case presented above had generalized anxiety disorder with comorbid alcohol abuse, we believe that the effectiveness of buspirone, considering the underlying mechanism of action, could also be generalized for treatment of anxiety symptoms in patients with underlying bleeding diathesis, even in the absence of substance use comorbidity, and thus could play a role as an important pharmacological treatment option in the treatment of anxiety symptoms for a subset of the patient population. Buspirone could be a safer anxiolytic alternative to benzodiazepines in patients with substance use disorders due to its apparent lack of significant concerns related to its addictive potential. As substance use could be seen as a “self-medication” strategy in some patients with untreated anxiety disorders increasing the risk for subsequent development of substance use disorders [8], successful pharmacological treatment of underlying anxiety symptoms could help with comorbid substance use as illustrated by the above case.
